# Structures of BRAF–MEK1–14-3-3 sheds light on drug discovery

**DOI:** 10.1038/s41392-019-0096-z

**Published:** 2019-12-13

**Authors:** Qiu Sun, Wenjing Wang

**Affiliations:** State Key Laboratory of Biotherapy, Cancer Center, West China Hospital, Sichuan University and Collaborative Innovation Center for Biotherapy, Chengdu, 610041 China

**Keywords:** Structural biology

In a recent paper published in *Nature*, Eunyoung Park et al. reported structures of autoinhibited and active BRAF–MEK1–14-3-3 complexes. This finding reveals the detailed mechanism underlying RAF regulation, which could facilitate the development of novel therapeutic strategies to overcome RAF-related cancer.^1^

The RAF/MEK/ERK signal transduction pathway regulates a wide set of cellular events, including cell proliferation, differentiation, and survival, via phosphorylation cascades in all eukaryotic cells.^2^ Previous studies have partially demonstrated the molecular mechanisms of the Raf/MEK/ERK cycle.^2^ Initially, GTP-bound RAS recruits Raf to the plasma membrane, which drives RAF to release inhibitory 14-3-3 protein from the N-terminus and form a dimer, thereby switching from an autoinhibited state to an activated state. Then, the activated Raf dimers enable MEK recruitment and phosphorylation, thereby transmitting the signal down to ERK. Finally, ERK signaling implements a negative feedback loop in which ERK phosphorylates several inhibitory sites in distinct regions of activated RAF, resulting in a release from the activated RAS and the disruption of RAF dimers. Some studies have revealed that BRAF and MEK are pre-associated in the quiescent state. In addition to its role in physiological processes, aberrant RAF activation in the key pathway contributes to causing cancer; thus, RAF kinase has been considered a target for anticancer treatment. Several Raf kinase inhibitors have been developed for clinical use and have achieved remarkable clinical outcomes. Nevertheless, most patients relapse within a year of treatment due to acquired resistance. Some RAF inhibitors unexpectedly promote RAF dimerization to induce the upregulation of ERK signaling, while some break the autoinhibitory status by disrupting the interactions of BRAF kinase with its N-terminal region.^3^ In the past decades, structures of isolated domains or fragments of the kinases targeting this pathway have been characterized to elucidate the mechanism of RAF/MEK/ERK^4–6^; however, the lack of a deeper structural characterization with intact structural information of RAF regulation has hindered the development of novel RAF/MEK/ERK kinase inhibitors.

To understand the normal regulation of RAFs, Park et al. recently reported the cryo-EM complex structures of full-length BRAF with MEK1 and 14-3-3 proteins in both the autoinhibited and active status.^1^ The mammalian homologs of RAF kinases (ARAF, BRAF, and CRAF) have three conserved regions called CR1, CR2, and CR3. CR1 at the N-terminal site contains the RBD (RAS binding domain) and the CRD (cysteine-rich domain) domain, the CR2 region consists of a binding site for the 14-3-3 protein, and CR3 at the C-terminal site has another binding site for the 14-3-3 protein, a phosphorylated motif and a serine/threonine kinase domain. The cryo-EM structure of an autoinhibited BRAF/MEK1^AA^/14-3-3 complex (MEK1^AA^: with alanine mutations at the phosphorylation sites of the activation segment) reveals the inhibitory mechanisms of the 14-3-3 dimer (PDB:6NYB). In an autoinhibited state, the active site of the BRAF kinase domain faces away from the 14-3-3 domain but faces MEK1 with extensive contact between the C-lobes of both kinases. ATP-γS is bound in the BRAF active site cleft, while ADP seems to be in a MEK active site. A 2.6 Å crystal structure of GDC-0623 and the ATP analog AMP-PNP-bound BRAF/MEK1^AA^ complex reveals high similarity with the cryo-EM structure (PDB:6PP9). A helix-like turn, called an inhibitory turn, stacks with hydrophobic residues in the glycine-rich loop and the β3 strand and the c-helix stabilize the inactive conformation. Rotation of the N-lobe results in a less open active site compared with other reported non-nucleotide bound BRAF structures, which implies the importance of ATP binding in the autoinhibited states. Two 14-3-3 proteins form a cradle-like space for the direct binding of the N-terminal CRD domain of BRAF, which blocks interface residues H510, D565, and Y566 for BRAF dimer formation and obstructs the CRD domain for membrane recruitment of BRAF and RAS-driven activation. The CRD domain is located in the center of the complex and exhibits β strands with two zinc ions stabilized at coordination sites. Two loops of the CRD domain (residues 239-245 and 253-260), which mediate the association of the domain with the membrane, extensively contact the highly conserved region of 14-3-3. As previously reported, R239 and T241 of BRAF are the key residues that bind 14-3-3. The pS365 and pS729 regulatory segments occupy the recognition groove on a separate side of the 14-3-3 dimer and interact with the kinase C-lobe and C-terminus of BRAF, respectively. The structure of the autoinhibited BRAF/MEK1^AA^/14-3-3 complex gives direct evidence that MEK also participates in stabilizing the inactive state of BRAF.

**Fig. 1:**
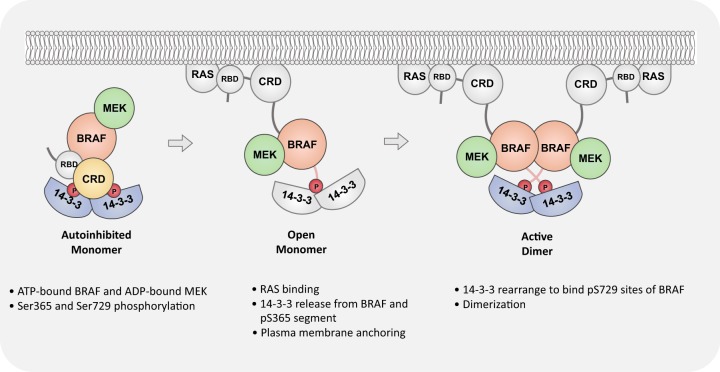
RAF activation model. The model depicts a possible mechanism for RAF activation from autoinhibited status to the active dimer. The 14-3-3 domain rearranges to bridge the pS729 site and promote dimer formation. The patterns in gray are modeled, whereas the others are structurally confirmed.

Furthermore, the BRAF^S365A^/MEK1^AA^/14-3-3 complex reveals a symmetrical back-to-back dimeric BRAF with MEK1 bound to each BRAF, while the 14-3-3 dimer rearranges to bridge the S729-phosphorylated tails of the two BRAFs in the active state (PDB: 6Q0J). BRAF S729 is stoichiometrically phosphorylated, but T599 and S602 exhibit negligible phosphorylation, although it is believed that autophosphorylation of T599 and S602 is critical for BRAF activation,^7^ which is consistent with the BRAF/14-3-3 complex. Interestingly, BRAF^S365A^/MEK1^AA^/14-3-3 with only one MEK bound to the BRAF dimer was observed under the same preparation conditions, which reveals an asymmetric position regarding the kinase dimer. The asymmetric structure indicates that 14-3-3 can regulate the affinity for MEK and control its release.

The structures of the BRAF–MEK1–14-3-3 complexes reveal an inactive and active conformation of BRAF, thereby providing mechanistic insights into RAF regulation. In the autoinhibitory state, the CRD and its membrane binding surface are mainly buried by the interaction with the 14-3-3 dimer and other fragments of RAF, whereas the RBD is exposed, allowing the activated RAS to recruit the stationary complex to the membrane. Upon RAS binding to RBD, BRAF kinase is released from 14-3-3 and forms an open monomer. Finally, the 14-3-3 domain rearranges to bridge the pS729 site of two RAF open monomers, thus promoting the formation of active RAF dimers (Fig. 1). Moreover, the study deepened the understanding of its activation by mutation or paradoxical activation by a RAF inhibitor, in which the autoinhibited state can be destabilized by weakening the interaction with ATP or disrupting the interaction with the inhibitory turn. Considering that an extensive interaction between ATP and BRAF in the autoinhibited state is observed, RAF inhibitors are more likely to promote conformational activation by replacing ATP from the quiescent state. Generally, detailed mechanistic studies of RAF activation and inhibitor pharmacology can promote the development of a novel RAF-related inhibitor design to overcome drug resistance in cancer therapy.
